# Pathogenesis, Host Resistance and Integrated Management of Sugarcane Smut Caused by *Sporisorium scitamineum*: A Comprehensive Review

**DOI:** 10.1111/mpp.70191

**Published:** 2025-12-17

**Authors:** Zhen Zeng, Qibin Wu, Dongjiao Wang, Wanying Zhao, Yuanyuan Zhang, Tingting Sun, Wankuan Shen, Youxiong Que

**Affiliations:** ^1^ State Key Laboratory of Tropical Crop Breeding Institute of Tropical Bioscience and Biotechnology, Sanya Research Institute, Chinese Academy of Tropical Agricultural Sciences Sanya Hainan China; ^2^ College of Agriculture, South China Agricultural University Guangdong Guangzhou China

**Keywords:** disease management, host resistance, multi‐omics, pathogenesis, *Sporisorium scitamineum*, sugarcane

## Abstract

Sugarcane smut, caused by the fungus *Sporisorium scitamineum*, is a devastating disease that threatens sugar production, leading to significant yield losses. The pathogen disrupts sugarcane growth by forming whip‐like structures filled with teliospores, which are disseminated through wind and rain. The control efforts are further complicated by its broad host adaptability, genetic diversity and ability to suppress host defences. Current management relies on resistant cultivars, agronomic practices, chemical and biological control measures, as well as emerging smart monitoring systems. Nevertheless, pathogen evolution and climate change pose ongoing challenges. This review synthesises recent advances in the biology, pathogenesis, host resistance mechanisms and integrated management strategies for sugarcane smut, with a particular focus on multi‐omics approaches that provide new insights into pathogen–host interactions. However, translating these insights into durable resistance and sustainable control strategies remains a key challenge for future research.

**Taxonomy:**

Domain Eukaryota, Kingdom Fungi, Phylum Basidiomycota, Class Ustilaginomycetes, Order Ustilaginales, Family Ustilaginaceae, Genus *Sporisorium*.

**Biology:**

*Sporisorium scitamineum* is a biotrophic fungus with a complex sexual‐asexual life cycle. Diploid teliospores germinate into haploid sporidia under favourable conditions. Compatible sporidia, regulated by tightly linked *a* and *b* mating‐type loci, mate to form dikaryotic hyphae, which invade sugarcane meristematic tissues via appressoria and cell wall degradation. The hyphae proliferate systemically in meristems and vascular tissues, divert host nutrients and finally induce whip‐like sori with new teliospores.

**Host Range:**

Primarily infects sugarcane; exhibits geographic genetic differentiation with adapted populations in Southeast Asia, South America and China. This regional adaptation is driven by long‐term host–pathogen co‐evolution and environmental factors.

**Disease Symptoms:**

Characterised by whip‐like sori at stalk apices, chlorotic and elongated leaves, increased tillering, thinner stalks and overall stunted growth, these symptoms collectively lead to reduced sugarcane yield and quality.

**Disease Control:**

Managed through resistant varieties, hot water treatment of seed cane, crop rotation, fungicides, biocontrol agents and smart systems using remote sensing and AI‐based forecasting.

## Graphical abstract

This pathogen profile explores sugarcane (*Saccharum* spp.) smut caused by *Sporisorium scitamineum*, highlighting advances in pathogen biology, molecular pathogenesis, host resistance and management

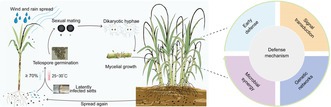

## 1 Introduction

Sugarcane (*Saccharum* spp.), a globally critical economic and energy crop, serves as the primary raw material for worldwide sugar production (Babu et al. 2022). It is predominantly cultivated in tropical and subtropical growing regions (Cavalett et al. 2012). Beyond its role in sugar supply, it is vital for rural employment, economic development, and food security (Liang et al. 2020). Global production is characterised by distinct regional dominance: Brazil leads, capitalising on its favourable climate, vast arable land and mature industry; India's production is deeply intertwined with its rural economy and domestic consumption; and China has established a highly regionalised and successfully upgraded production hub centered in Guangxi and Yunnan provinces (Guga et al. 2021; Joshi et al. 2024; Soltangheisi et al. 2019).

Sugarcane smut, caused by *Sporisorium scitamineum*, poses a major threat to global production (Que, Xu, et al. 2014). The genetic diversity and geographical differentiation of *S. scitamineum* populations are intrinsically linked to regional variations in planting patterns and environments, reflecting the pathogen's long‐term adaptation and underscoring the need for a systematic understanding of its biology and control (Dutheil et al. 2016). Advances in multi‐omics technologies are now enabling deeper insights into its pathogenesis, host resistance and sustainable management strategies, forming the core rationale for this review.

Our review highlights the key findings on *S. scitamineum* life cycle, infection mechanism and host resistance, emphasising the role of multi‐omics in uncovering the pathogen's genetic diversity and evolving resistance mechanisms. Specifically, we examine the correlation between pathogen genetic differentiation and the global sugarcane landscape. Besides, we dissect the molecular mechanisms of pathogen–host interaction and highlight the application of multi‐omics technologies in resistance research. Concurrently, knowledge gaps and core challenges in disease management are identified, while future research directions are outlined. This review aims to deepen the understanding of sugarcane smut pathogenesis and provide theoretical support for building efficient and sustainable control systems.

## 2 Biology and Epidemiology

### 2.1 Life Cycle and Infection Process

**FIGURE 1:**
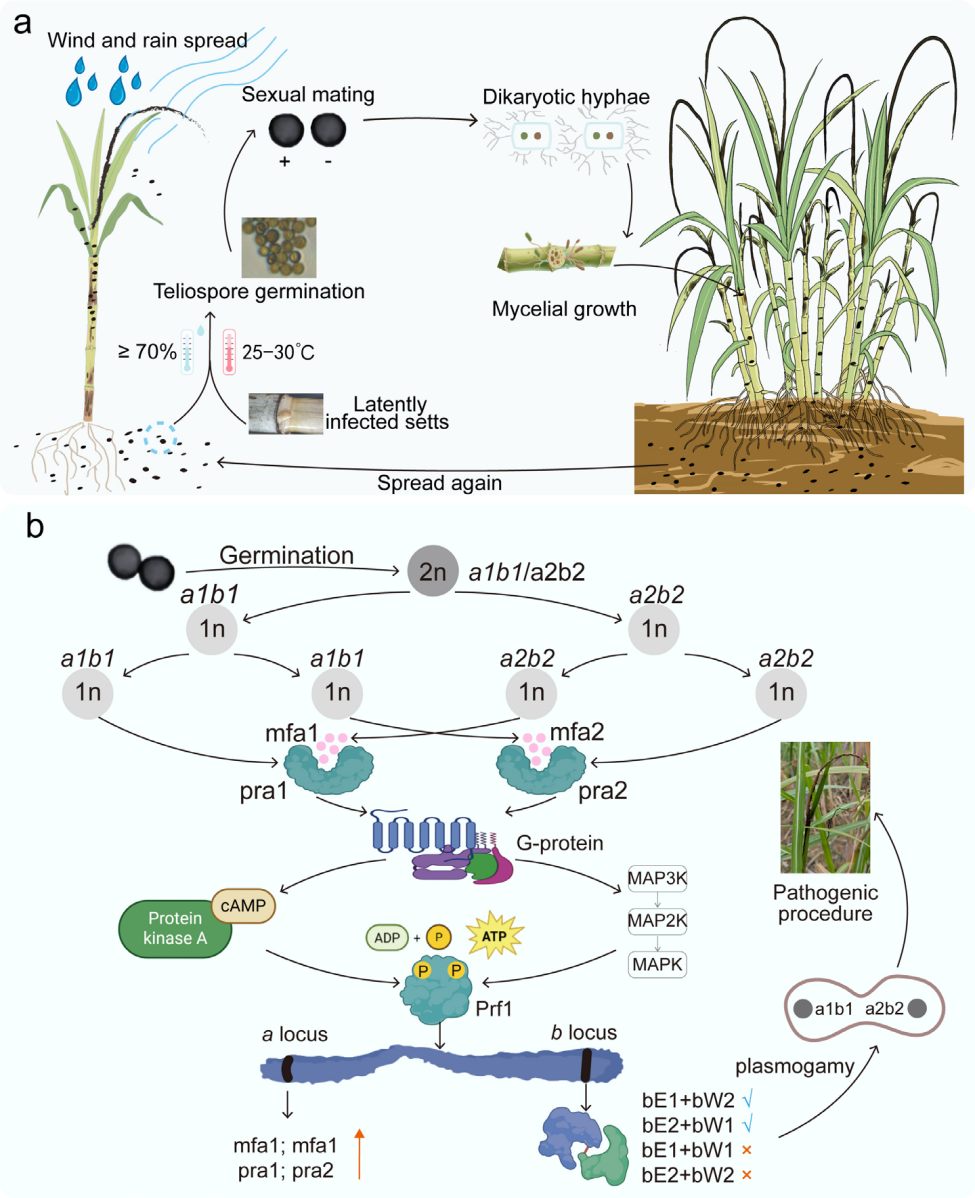
Life cycle of *Sporisorium scitamineum* from spore germination to whip formation in sugarcane. (a) The disease cycle and epidemiological drivers of sugarcane smut. The cycle begins with teliospores, the dormant diploid spores, present in soil or on planting material (setts). Under favourable conditions, these spores germinate and undergo meiosis to produce haploid, yeast‐like sporidia of compatible mating types (+ and −). Following mating, the resulting dikaryotic hyphae infect the sugarcane plant, primarily through buds. The fungus grows systemically within the plant, eventually inducing the formation of a characteristic whip‐like sorus, which is full of new teliospores. These spores are disseminated by wind and rain, completing the cycle. The three key epidemiological components, comprising environmental conditions (e.g., temperature, humidity), host management (e.g., variety susceptibility, sett quality) and transmission pathways, govern the severity and spread of the disease. (b) Molecular events during mating and pathogenicity initiation. Diploid teliospores germinate and undergo meiosis to produce haploid basidiospores of two mating types (*a1b1* and *a2b2*), determined by the tightly linked *a* and *b* loci. Recognition between adjacent cells of compatible mating types is mediated by pheromone‐receptor pairs (e.g., Mfa1‐Pra2), triggering intracellular G protein and cAMP/MAPK signalling cascades. This leads to phosphorylation and activation of the transcription factor Prf1. Prf1 drives the expression of genes for mating, filamentous growth and pathogenicity. A critical step is the formation of an active heterodimeric complex between the bE and bW homeodomain proteins from different alleles, which serves as the master regulator establishing the stable, infectious dikaryon capable of host invasion.

Early sugarcane smut infections are often asymptomatic, and their reliable detection typically depends on molecular assays. Consequently, infected plants are frequently overlooked, leading to significant pathogen accumulation within fields and exacerbating disease (Rajput et al. 2021). For newly planted cane, primary inocula include teliospores (dormant spores) in the soil and fungal mycelium within infected seed cane (Hoy et al. 1991; Vicente et al. 2021). Mycelium from infected seed cane invades the meristematic tissue (growing point) of stalks via plasmodesmata. Soil‐borne teliospores germinate under conducive environmental conditions, producing haploid sporidia. Compatible sporidia then fuse to form dikaryotic hyphae, which also invade the growing point. Following invasion, hyphae proliferate systemically within the plant, absorbing nutrients and ultimately inducing the smut whip at the stalk apex (Su et al. 2013). This whip‐like structure comprises dense masses of teliospores (Fontaniella et al. 2002). Upon maturity, these spores disperse via wind or rain to infect new plants, thereby perpetuating the disease cycle (Figure 1a). Infected plants exhibit various symptoms, including increased tillering, thinner stalks, chlorotic and elongated leaves and stiff upright tops, all of which collectively severely impair growth and reduce yield (Rajput et al. 2021; Taniguti et al. 2015).

### 2.2 Disease Epidemiology

Sugarcane smut epidemics are driven by multiple interacting factors. Environmental conditions, particularly temperature, humidity and water stress, are primary determinants of infection efficiency by influencing pathogen survival and virulence (Croft and Braithwaite 2006; Villajuana‐Bonequi et al. 2019). For instance, teliospore germination typically occurs between 25°C and 30°C and requires relative humidity exceeding 70% (Figure 1a). Host factors, especially varietal resistance, are equally critical (Ferreira et al. 1989). Agronomic practices also substantially influence disease risk. For instance, fields under continuous ratoon cropping for 3 years accumulate significantly higher soil teliospore loads than newly planted fields (Bhuiyan et al. 2021). Furthermore, close planting (row spacing < 1.2 m) elevates canopy humidity, creating conditions favourable for spore germination and consequently increasing disease incidence. Infected seed cane serves as the primary vehicle for cross‐regional dissemination, whereas wind‐mediated spore dispersal exhibits distance‐dependent decay (Bhuiyan et al. 2021). A particularly concerning factor is the synergistic interaction with insect vectors (Viswanathan 2020).

### 2.3 Taxonomy and Genetic Diversity

*Sporisorium scitamineum*, a significant plant‐pathogenic fungus belonging to the order Ustilaginales (Basidiomycota), occupies a core evolutionary clade within the Ustilaginaceae family based on molecular phylogenetics, showing closest kinship to *Ustilago maydis* (Begerow et al. 2006; Piepenbring et al. 2002). Whole‐genome sequencing reveals the rich transposable elements (TEs) in its genome. These active mobile elements constitute a crucial foundation for rapid genetic variation and adaptive evolution, enabling the pathogen to cope with environmental pressures and host resistance (Dutheil et al. 2016). Population genetics studies demonstrate significant intraspecific genetic diversity. There are distinct population structures with strong geographical isolation (Xu et al. 2014). Globally, populations cluster primarily into three major groups: Southeast Asia (including India, Thailand), South America, and a relatively distinct Chinese group (represented by major producing regions such as Guangxi and Yunnan) (Bhuiyan et al. 2021; Singh et al. 2005). This differentiation reflects long‐term regional adaptation and evolution.

## 3 Pathogenesis

### 3.1 Genomic Basis of *S. scitamineum* Pathogenicity

#### 3.1.1 Mating Type and Pathogenicity Regulation

Pathogenicity initiation in *S. scitamineum* begins with the germination of diploid teliospores. Following meiosis, these spores produce four haploid basidiospores. Mating compatibility is governed by the *a* and *b* mating‐type loci (Vollmeister et al. 2012; Wahl et al. 2010). *S. scitamineum* exhibits tight linkage of both mating‐type loci on the same chromosome, unlike most tetrapolar basidiomycetes such as *U. maydis* , in which these loci are located on different chromosomes (Djamei and Kahmann 2012; Zhang et al. 2019). As a result, meiotic recombination rates are low (< 5%), leading to basidiospores that typically harbour only two complementary mating types, *a1b1* and *a2b2* (Piepenbring et al. 2002; Yan, Dai, et al. 2016). The spatial proximity of these compatible types further facilitates efficient mating (Figure 1b).

Mating initiation between adjacent cells of differing types relies on a highly specific recognition system encoded by the *a* locus: Mfa1 pheromone secreted by *a1* cells binds the Pra2 receptor on *a2* cells, while Mfa2 from *a2* cells binds Pra1 on *a1* cells (Yan, Zhu, et al. 2016). This strict Mfa1‐Pra2/Mfa2‐Pra1 interaction triggers G protein dissociation, thereby activating conserved cAMP and MAPK signalling cascades (Chang et al. 2019). Ultimately, these converging signals induce dual phosphorylation of the transcription factor Prf1 at critical sites (Catlett Natalie et al. 2003). Phosphorylated Prf1 translocates to the nucleus and activates transcription at both the *a* and *b* loci (Cai et al. 2021). Subsequently, activation of the *a* locus establishes a positive feedback loop amplifying pheromone signalling. Simultaneously, *b* locus activation expresses the homeodomain transcription factors bE and bW. Additionally, Prf1 directly induces expression of certain pathogenicity‐related genes and orchestrates the cellular morphogenesis required for mating cell fusion (Figure 1b). After the fusion of compatible haploid cells, the expressed bE and bW proteins interact. Notably, stable and functional heterodimer formation requires bE and bW proteins to originate from different allelic variants (Agisha, Nalayeni, et al. 2022). Acting as the master regulatory complex, this heterodimer orchestrates dikaryotic mycelium formation and maintenance while also activating the pathogenic gene expression programme (Taniguti et al. 2015). It is this invasive dikaryotic mycelium that penetrates sugarcane tissues, disrupts host physiology, and ultimately drives smut symptom development (Figure 1b).

#### 3.1.2 Evolution of Effector Protein‐Encoding Genes

Pathogenic adaptability in *S. scitamineum* is fundamentally linked to effector gene evolution. There are significantly more numerous and larger clusters of candidate secreted effector proteins in *S. scitamineum* than other smut fungi (Dutheil et al. 2016). In this species, tandem gene duplication serves as the primary driver for cluster evolution, a process accelerated by TE activity. Genomic compartmentalisation confines TE activity to specific regions, and this spatial restriction potentially allows adaptive TE influences near effectors under positive selection, while concurrently shielding the core genome from deleterious TE effects (Faino et al. 2016). Interestingly, high sequence relatedness within clusters strongly supports tandem duplication as the principal formation mechanism (Dutheil et al. 2016). Furthermore, effector genes, especially a substantial proportion of orphan effectors lacking identifiable homologues, experience positive selection pressure (Dutheil et al. 2016). These orphan genes are likely to undergo rapid evolution to counteract host resistance, thereby enhancing the adaptive fitness of the pathogen.

#### 3.1.3 Evolution of Secreted Substances and Pathogenic Factors

Closely reflecting host adaptation, the evolution of the secretome of *S. scitamineum* exhibits high synergy with its pathogenic strategy. This secretome encompasses cell wall‐degrading enzymes (CWDEs), redox enzymes and signalling molecules (Schuster et al. 2018). For instance, secreted arginase functions as a key quorum‐sensing (QS) factor, inducing cellular aggregation that is essential for effective invasion (Que, Su, et al. 2014; Sánchez‐Elordi et al. 2015). Glycosylation modifications of this enzyme enhance its binding affinity to host receptors, representing an evolutionary adaptation of a key pathogenic co‐factor (Millanes et al. 2008). Additionally, secreted redox enzymes, such as glutathione S‐transferases (GSTs), scavenge host‐derived reactive oxygen species (ROS) to augment pathogenicity. To counter host oxidative defences effectively, genes encoding these enzymes have undergone duplication events, increasing copy number and expression levels (Faino et al. 2016).

### 3.2 Infection Mechanism and Molecular Strategy of *S. scitamineum*

#### 3.2.1 Spore Germination and Host Signal Perception

The invasion of *S. scitamineum* is dependent on the synergistic action of QS and host‐derived signals (Galloway et al. 2012). Upon reaching the host surface, teliospores secrete QS signal molecules or specific hydrolytic enzymes. These molecules bind to receptors on the spore wall, thereby triggering cellular aggregation. Once aggregation achieves a critical density, signalling molecule concentration becomes sufficient to induce changes in gene expression (Yan, Dai, et al. 2016). These alterations include upregulation of metabolic enzymes like arginase, which accumulate the energy and infection units required for germination and host invasion (Sánchez‐Elordi et al. 2015). Furthermore, *S. scitamineum* perceives physical and chemical cues on the host surface, including plant cuticle hydrophobicity and cutin monomers. Such host signals induce appressoria formation. This signal perception likely involves transmembrane receptors activating downstream pathways to ultimately drive expression of infection‐essential genes (Dutheil et al. 2016).

#### 3.2.2 Cell Wall Degradation and Tissue Penetration

*Sporisorium scitamineum* breaches the physical barriers of the host primarily through the secretion of CWDEs. The secretome of *S. scitamineum* contains a diverse array of hydrolytic enzymes capable of degrading key polysaccharide components of the plant cell wall, such as xylan and arabinan (Kubicek et al. 2014; Ma et al. 2018; Xia et al. 2020). During the penetration process, the pathogen inevitably encounters the constitutive structural defences of the host. To circumvent these defences and potentially avoid host recognition, *S. scitamineum* appears to regulate the expression of its CWDE arsenal strategically. For instance, the expression of certain CWDEs is subject to catabolite repression control under conditions of abundant carbon sources. This minimises enzyme production when readily available carbon is present, thereby reducing the likelihood of the host detecting and responding to active cell wall degradation (Huang et al. 2025; Nair and Sarma 2021; Xia et al. 2020).

#### 3.2.3 Hyphal Expansion and Nutrient Acquisition

*Sporisorium scitamineum* exhibits a biotrophic lifestyle. Its mycelia expand systemically around host parenchyma cells and vascular tissues (Su et al. 2013). During expansion, mycelia preferentially colonise metabolically active meristems to ensure continuous nutrient supply, moving intercellularly and acquiring nutrients from living cells via biotrophic interfaces (Gebrie 2016; Que, Su, et al. 2014). To secure nutrients, the pathogen induces host metabolic reprogramming, triggering a source–sink shift that transforms the infection site into a nutrient sink. The pathogen then regulates host sucrose transport and metabolism genes, upregulating soluble acid invertase genes to promote sucrose hydrolysis into hexoses (carbon source), while inhibiting starch synthesis genes to reduce carbohydrate storage and enhance nutrient availability (Djamei and Kahmann 2012; Horst et al. 2010).

### 3.3 Molecular Strategies Employed by the Pathogen to Suppress Host Defence Responses

#### 3.3.1 Suppression of Cell Wall Reinforcement

**FIGURE 2:**
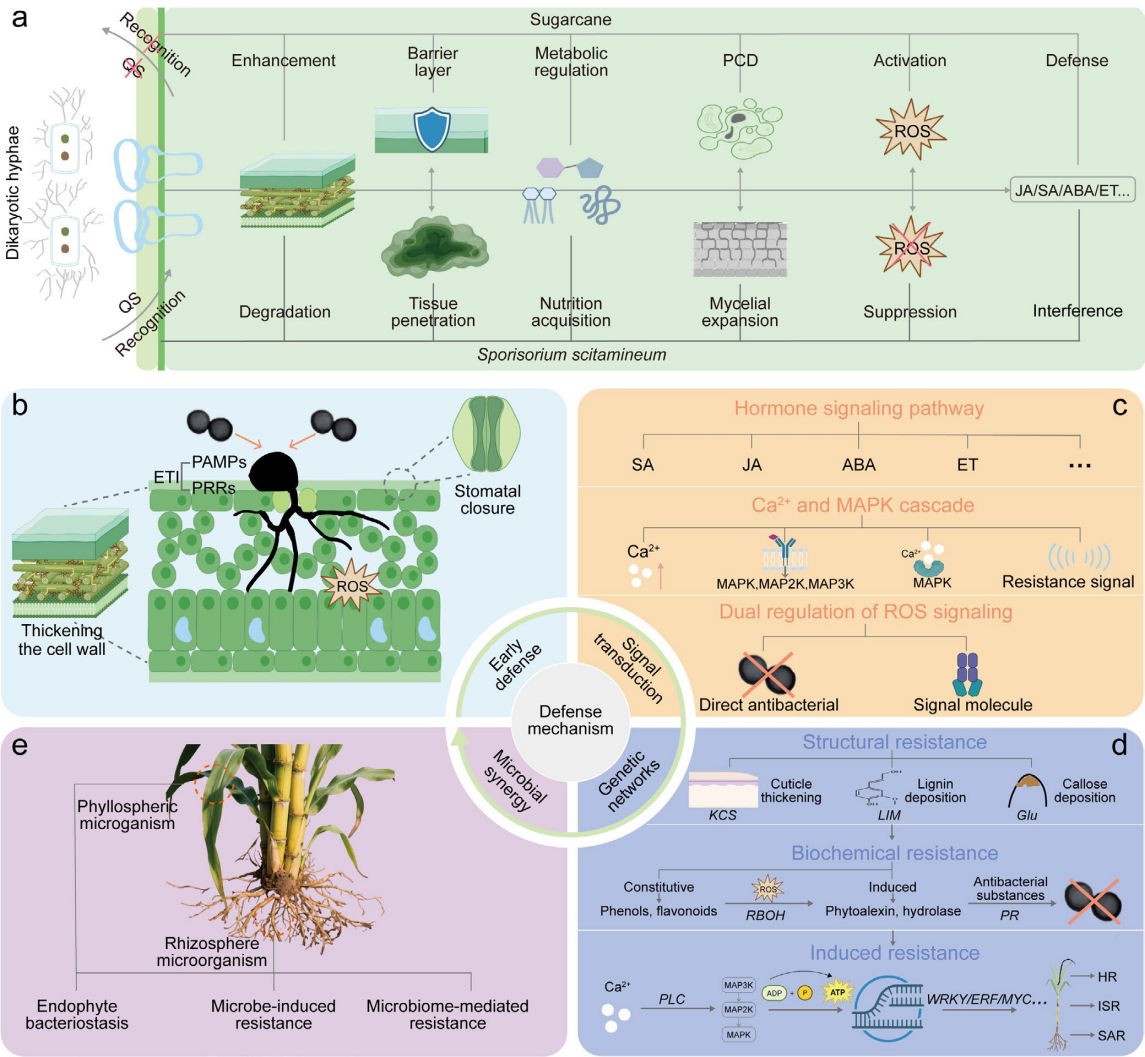
The battle between sugarcane and smut fungus: invasion strategies and plant defence responses. (a) The invasion mechanisms of the smut fungus and the corresponding basal defence responses of sugarcane. The diagram illustrates the key stages of interaction at the infection site. The fungus perceives plant surface signals to differentiate appressoria for penetration, using quorum sensing (QS) and cell wall‐degrading enzymes (CWDEs). Inside the plant, it proliferates and employs various virulence strategies, such as secreting effector proteins to suppress host reactive oxygen species (ROS) bursts and programmed cell death (PCD), interfering with defence hormone signalling (e.g., salicylic acid [SA], jasmonic acid [JA], abscisic acid [ABA], ethylene [ET]), and blocking host cell wall reinforcement. In response, sugarcane activates pattern‐triggered immunity (PTI) upon recognition of pathogen‐associated molecular patterns (PAMPs). This basal defence includes rapid cell wall lignification, a defensive oxidative burst, activation of hormone‐mediated defence pathways, and the production of antimicrobial compounds via the phenylpropanoid pathway. (b–e) The multilayered defence system of sugarcane against smut. The resistance of the plant integrates several coordinated layers: (b) Early physical and chemical barriers, including PAMP recognition (PAMP‐triggered immunity [PTI]) by pattern recognition receptors (PRRs), cell wall reinforcement, and accumulation of preformed and induced antimicrobial secondary metabolites. (c) Intricate signal transduction networks involving hormones, calcium ions (Ca^2+^), mitogen‐activated protein kinase (MAPK) cascades and ROS signalling for precise defence signal amplification and coordination. (d) Core regulatory mechanisms encompassing transcriptional and post‐transcriptional control of defence genes, metabolic reprogramming to fuel defence pathways (e.g., phenylpropanoid metabolism) and careful management of ROS and energy homeostasis. HR, hypersensitive response; ISR, induced systemic resistance; SAR, systemic acquired resistance. (e) Synergistic contributions of the plant microbiota, which enhance host resistance through direct antagonism of the pathogen, ISR and maintenance of a protective microecological balance.

The plant cell wall serves as the primary physical barrier against pathogen invasion. To overcome this, *S. scitamineum* employs multiple strategies to inhibit host cell wall reinforcement (Figure 2a). Crucially, it secretes effector proteins, such as the highly conserved (among smut fungi) Cce1, which directly suppresses callose deposition. Callose acts as a key physical barrier that is rapidly synthesised at attempted penetration sites, and that is why Cce1‐mediated inhibition can prevent its formation. Loss of Cce1 function results in early blockage of the pathogen by the host (Hemetsberger et al. 2015; Seitner et al. 2018). Concurrently, the fungus likely diminishes host lignin synthesis through metabolic diversion, reducing cell wall mechanical strength. Furthermore, to counter increased phenolic compounds (e.g., ferulic acid, caffeic acid) in resistant varieties, which are compounds that promote lignin cross‐linking, *S. scitamineum* potentially secretes esterases or oxidases to degrade these phenolics, disrupting their structural reinforcement role (Jose et al. 2017).

#### 3.3.2 Suppression of ROS Burst

The rapid production of ROS constitutes a pivotal early host defence response during plant–pathogen interactions. *S. scitamineum* has evolved specific countermeasures (Figure 2a). Immediate suppression is achieved through secreted effectors that directly interfere with host ROS metabolism. For instance, the effector Pep1 specifically inhibits sugarcane peroxidase (POD), which are key enzymes responsible for generating ROS like H_2_O_2_. By inhibiting POD, the pathogen reduces ROS accumulation, thus minimising oxidative damage to its hyphae (Hemetsberger et al. 2015). Additionally, the pathogen enhances its intrinsic ROS tolerance by manipulating the antioxidant system of the host. Following infection, the expression of host antioxidant enzymes, such as glutathione S‐transferases (GSTs), is significantly upregulated. These enzymes scavenge excess ROS, preventing the oxidative burst of the host from exerting toxic pressure on the pathogen and thereby facilitating colonisation (Peters et al. 2017).

#### 3.3.3 Interference With Hormone Signalling Pathways

Plant hormones play a central role in orchestrating defence responses. *S. scitamineum* actively disrupts host hormone signalling to attenuate defences, with the salicylic acid (SA) pathway being a primary target (Figure 2a). A key strategy involves the potential secretion of effector proteins that mimic enzymes like chorismate mutase. Such effectors could catalyse the conversion of host chorismate into prephenate, diverting this metabolic precursor away from SA biosynthesis (Djamei et al. 2011; Xia et al. 2020). Reduced SA accumulation in turn suppresses the expression of SA‐mediated defence genes, including pathogenesis‐related (PR) genes. Moreover, effectors interfere with the signalling balance between jasmonic acid (JA) and ethylene (ET). For example, the downregulation of the JA pathway observed in susceptible varieties may compromise the synergistic defence actions often coordinated between JA/ET and SA pathways, further weakening the integrated immune response of the host (Agisha, Ashwin, et al. 2022; Que, Su, et al. 2014).

## 4 Resistance Mechanisms

### 4.1 Pathogen Recognition and Early Defence Initiation

Early resistance in sugarcane to smut fungus begins with the recognition of conserved molecular patterns from the pathogen. This recognition then triggers the rapid initiation of defence responses, which include both physical and chemical defences.

#### 4.1.1 Pathogen‐Associated Molecular Pattern‐Triggered Immunity

Pattern recognition receptors (PRRs) on the sugarcane cell membrane are central to this recognition. Based on knowledge from other plants, chitin receptor complexes containing LysM domains are strong candidates for recognising chitin oligomers in sugarcane as well (Shinya et al. 2015). However, key details in sugarcane remain unknown. These include the exact composition and regulation of these receptor complexes. Furthermore, it is still an active research topic whether these receptors are effective against the full range of pathogen‐associated molecular patterns (PAMPs) present in the smut fungus (Makechemu et al. 2025). This recognition activates the intracellular kinase domain of the receptor, which initiates the MAPK signalling cascade. The activated MAPKs then phosphorylate transcription factors. This phosphorylation modifies the activity of transcription factors, enabling them to translocate to the nucleus and initiate the expression of downstream defence genes. This process establishes a basal layer of resistance (PAMP‐triggered imminity, PTI) against the smut fungus (de Paula et al. 2018; Huang et al. 2018; Lu et al. 2021).

#### 4.1.2 Reinforcement of Physical Barriers

The smut fungus primarily invades through epidermal cell gaps (or intercellular spaces) in young sugarcane tissues. Physical defence in sugarcane involves dynamic restructuring of cell walls, regulation of plasmodesmata, and accumulation of silica granules to impede invasion and spread (Figure 2b). Cell wall thickening exhibits a ‘zoned deposition pattern’. Cells directly at the infection site thicken rapidly and substantially. This reinforcement then gradually diminishes in the surrounding cells. This pattern effectively forms a physical barrier that impedes hyphal extension (Chen et al. 2025; Marques et al. 2018). Furthermore, sugarcane precisely regulates plasmodesmatal aperture to prevent hyphal spread. Upon infection, the microtubule cytoskeleton facilitates the aggregation of callose synthase at the plasmodesmatal orifices. This enzyme synthesises callose plugs that reduce the effective plasmodesmatal pore size. The resulting diameter is significantly smaller than that of the invading hyphae, thus blocking their cell‐to‐cell movement. Simultaneously, plasmodesmata‐localised proteins may be phosphorylated. This phosphorylation enables them to recruit actin microfilaments, which then surround the plasmodesmata. This action further constricts the aperture and enhances the isolation of infected cells (Brunkard and Zambryski 2017).

#### 4.1.3 Chemical Defence: Antimicrobial Action of Secondary Metabolites

Sugarcane employs a ‘basal storage + induced synthesis’ strategy for secondary metabolites to inhibit key life cycle stages of the smut fungus (Figure 2b). Phenolic compounds like chlorogenic acid and quercetin, stored in vacuoles prior to infection, are rapidly released upon invasion. Concurrently, the phenylpropanoid pathway is highly upregulated, leading to the synthesis of defence compounds such as coumarins and flavonoids (Chen et al. 2024). Post‐infection, secondary metabolism undergoes significant reprogramming. The expression of key enzymes, such as chalcone synthase, is increased, promoting the synthesis of flavonoids (e.g., luteolin) that inhibit appressorium formation. Concurrently, enzymes driving lignin precursor synthesis are activated, reinforcing cell walls (Gleeson and Sheedy 2016). Additionally, sugarcane accumulates antimicrobial terpenoid compounds at the later stages of infection. These compounds are thought to target the fungal cell membrane, cytoskeleton or key metabolic pathways (Cheng et al. 2011). Alkaloids are synthesised later, accumulating to form a barrier in vascular bundles or apical meristem regions, hindering systemic spread towards the apical bud and reducing whip formation (Shi et al. 2022).

### 4.2 Signal Transduction Pathways: Transmission and Amplification of Resistance Signals

Infection signals from smut fungus require transmission and amplification through complex signalling networks to coordinate whole‐plant defences (Figure 2c).

#### 4.2.1 Synergy and Antagonism in Hormone Signalling Pathways

The immune response of sugarcane to smut disease relies on a hormonal regulatory network, with core components including SA, JA, abscisic acid (ABA), ethylene (ET), auxin (IAA), cytokinin (CK) and gibberellin (GA). These hormones interact via synergy, antagonism and spatiotemporal specificity to coordinate effective defence.

SA is a core defence signal against the biotrophic smut fungus (Zeng et al. 2025). After accumulation, SA triggers a series of changes in the NPR1 protein: it causes NPR1 to depolymerise, become phosphorylated, and move into the nucleus. Once inside the nucleus, NPR1 binds to TGA‐type transcription factors. This binding activates the expression of defence genes, such as PR1 and PR5, leading to the establishment of systemic acquired resistance (SAR) (Zang et al. 2025). JA signalling plays a crucial complementary role: bioactive JA‐Ile triggers degradation of JAZ repressors, releasing transcription factor MYC2 to induce downstream defence genes and stimulate defensive phytoalexins (e.g., terpenoids). A tissue‐specific antagonism between SA and JA is frequently observed. SA dominates in stalks (the primary infection sites), while JA is more prominent in leaves (Sun et al. 2023). However, this model may be an oversimplification. Evidence also exists for context‐dependent synergy between these hormones. The precise spatiotemporal dynamics of these hormones during infection are not yet fully understood. ABA generally suppresses resistance to biotrophs; in sugarcane, models propose that resistance mechanisms attenuate ABA signalling (Martins et al. 2024). This hypothesis is plausible but requires experimental confirmation, like quantifying ABA flux or validating key ABA metabolic genes in the sugarcane–smut interaction.

ET signalling acts synergistically in the defence network: activated ethylene response factors (e.g., EIN3/EIL1) regulate defence genes involved in cell wall reinforcement and PR protein synthesis. ET shows significant synergy with JA to promote downstream defences and can positively regulate SA pathway components, enhancing overall defence (Chen et al. 2021). IAA, CK and GA play context‐dependent roles: sugarcane may induce *GH3* family genes (to conjugate and inactivate IAA) and regulate auxin transporters to reduce IAA at infection sites (Nagarajan et al. 2023). CK signalling, via ARR transcription factors, positively regulates certain SA pathway genes and antioxidant defences, and may support SA‐mediated defence by antagonising ABA. GA signalling operates via DELLA repressors; that is, DELLAs interact with JAZ repressors or enhance MYC2 activity in the JA pathway, promoting JA‐responsive gene expression and defence compound synthesis (Bhuiyan et al. 2021).

#### 4.2.2 Calcium Signalling and MAPK Cascades

Calcium signalling is an early event in the perception of smut infection in sugarcane (Zhang et al. 2024). The smut fungus or its derived elicitors trigger the opening of plasma membrane calcium channels, resulting in an initial influx of Ca^2+^. This is followed by the release of Ca^2+^ from intracellular stores, generating distinct oscillatory calcium signatures. The MAPK cascade acts as a major downstream pathway transducing these calcium signals into defence gene activation. Elevated cytosolic Ca^2+^ can stimulate MAPKKKs. These activated MAPKKKs then phosphorylate and activate the next level of kinases, called MPKs. Once active, the MPKs perform two key functions: they regulate the expression of defence‐related genes and they promote the synthesis of secondary metabolites. Additionally, MPKs may provide feedback regulation on calcium channel activity, forming synergistic signal amplification loops together with Ca^2+^‐activated CDPKs.

#### 4.2.3 Dual Regulation of ROS Signalling

ROS play a dual role in smut resistance, serving as both direct antimicrobial toxins and defence signalling molecules. This necessitates a precise balance between ROS production and scavenging (Wang, Gou, et al. 2025; Wu, Chen, et al. 2023). Early in infection, plasma membrane NADPH oxidases are activated, often via Ca^2+^ signalling, to produce a localised ROS burst that inhibits appressorium formation and alerts adjacent cells. The antioxidant system tightly regulates ROS levels. Initial excess H_2_O_2_ is rapidly scavenged by enzymes like superoxide dismutase (SOD), POD and catalase (CAT) to prevent self‐damage. As infection progresses, systems like the glutathione‐ascorbate cycle maintain subtle ROS for signalling (Chen et al. 2024). Later, ROS are specifically cleared from critical organelles like mitochondria to ensure energy for defence. ROS signalling operates through redox‐sensitive proteins. H_2_O_2_ oxidatively modifies these proteins, leading to the activation of transcription factors like NPR1 and TGAs, which induce defence gene expression. Spatial H_2_O_2_ gradients at infection sites are also thought to guide the directional transport of defence compounds.

### 4.3 Expression Regulation of Disease Resistance‐Related Genes

The core of the resistance mechanism of sugarcane to smut lies in the precise regulation of disease resistance‐related gene expression (Figure 2d). In structural resistance, gene regulation enables physical barrier formation. Resistant varieties activate specific transcription factors to upregulate phenylpropanoid pathway key genes (*LIM*), promoting massive lignin accumulation in cell walls for mechanical defence (Laksana et al. 2024). Early infection induces β‐1,3‐glucan synthase gene expression, stimulating callose deposition in intercellular spaces to block hyphal extension (Su, Wang, et al. 2016). For epidermal reinforcement, cutin/wax synthesis genes (*KCS*) are modulated, thickening wax layers and enhancing cutin cross‐linking to reduce pathogen penetration (Li et al. 2024).

Biochemical resistance also depends on gene regulation. Constitutive resistance relies on high, steady expression of flavonoid synthase genes, plus genes encoding cysteine‐rich peptides and chitinases/β‐1,3‐glucanases (Su, Wang, et al. 2016). Inducible resistance involves dynamic regulation: pathogen recognition induces NADPH oxidase genes for ROS bursts, while antioxidant enzyme genes (SOD, POD, CAT) are co‐induced/differentially regulated to maintain ROS homeostasis (Chen et al. 2024). Post‐infection, chitinase/β‐1,3‐glucanase genes are strongly induced to degrade pathogen cell walls. Defence gene expression is finely controlled by positive and negative regulators. Specific WRKY transcription factors may act as negative regulators suppressing PR protein or defence metabolite synthesis genes (Wang, Gou, et al. 2025), while activated MAPKs phosphorylate JA/ET pathway components to induce defence genes.

Gene regulation is also essential for induced resistance signal perception and transduction. Pathogen effectors alter plasma membrane calcium channel gene activity/expression, increasing cytosolic Ca^2+^ (Wu, Zhang, et al. 2024); susceptible plants highly express specific Ca^2+^ transporters to terminate signalling, while disrupting these transporters prolongs defence. In phospholipid cascades, specific transfer/sensing proteins activate phospholipase C (generating IP_3_ and DAG), which induces calmodulin/calmodulin‐like protein genes and MAPK cascade genes, finally activating systemic defence genes (Wu, Zhang, et al. 2024).

### 4.4 Plant–Microbe Interactions: Synergistic Defence by Symbiotic Microbes

Resistance in sugarcane to smut relies on its own mechanisms and also on crucial synergistic relationships with symbiotic microbes in the rhizosphere, endosphere and phyllosphere (Figure 2e).

#### 4.4.1 Direct Antimicrobial Action by Microbes in Different Niches

Symbiotic microbes directly inhibit the smut fungus through antimicrobial compound secretion, resource competition, or spatial occupation, with strategies varying across niches. Endophytic and rhizospheric microbes primarily function via ‘chemical attack’ and ‘resource competition’. For instance, endophytic *Bacillus* spp. secretes antimicrobial compounds like lipopeptides (e.g., surfactin, iturin) and polyketides, which disrupt fungal membranes and inhibit spore germination and hyphal growth. They produce chitinases and β‐1,3‐glucanases that synergise with their own PR proteins of sugarcane to degrade the fungal cell wall (Zheng et al. 2021).

#### 4.4.2 Microbial Induction and Priming of Host Defences

Symbiotic microbes act as elicitors via their molecular patterns or metabolites, triggering sugarcane defence responses. This often results in induced systemic resistance (ISR) or localised priming, with different microbial groups activating distinct signalling pathways (Van Wees et al. 2008). For example, endophytic and rhizospheric microbes frequently elicit whole‐plant resistance. Their components, like peptidoglycan fragments from actinomycetes, are recognised by sugarcane immune receptors. This activates SA or JA/ET pathways, inducing systemic defence gene expression and establishing a primed state (Mehdi et al. 2025; Stacy et al. 2021). In contrast, arbuscular mycorrhizal fungi colonise roots and transmit signals to shoots, mainly activating JA/ET pathways to promote phytoalexin synthesis and silicon accumulation. Meanwhile, phyllospheric microbes specialise in localised pre‐defence. Metabolites from fungi like *Aspergillus* spp. induce cytosolic Ca^2+^ elevation in plant cells. This activates CDPK and MAPK cascades, which not only trigger local defences but may also sharpen the host's ability to detect pathogen PAMPs (Mhlongo et al. 2018).

#### 4.4.3 Disease Resistance Mediated by Microbial Community Structure and Function

The composition, balance and diversity of rhizospheric, phyllospheric and endophytic microbial communities are fundamental to long‐term sugarcane smut resistance. Studies have shown that healthy sugarcane harbours microbial communities in these niches enriched with biocontrol‐beneficial bacteria (e.g., *Pseudomonas*, *Bacillus*), while diseased plants have reduced beneficial bacteria and increased potential pathogens/saprophytes (e.g., *Fusarium* spp.) (Rocha et al. 2021; Shair et al. 2021). Sugarcane actively modulates its microbiome via root exudates (e.g., phenolic acids, organic acids) and leaf exudates (e.g., specific sugars, volatiles), which selectively promote beneficial microbe colonisation and inhibit pathogens, maintaining a disease‐suppressive community.

### 4.5 Application of Multi‐Omics Technologies in Resistance Mechanism Research

**FIGURE 3:**
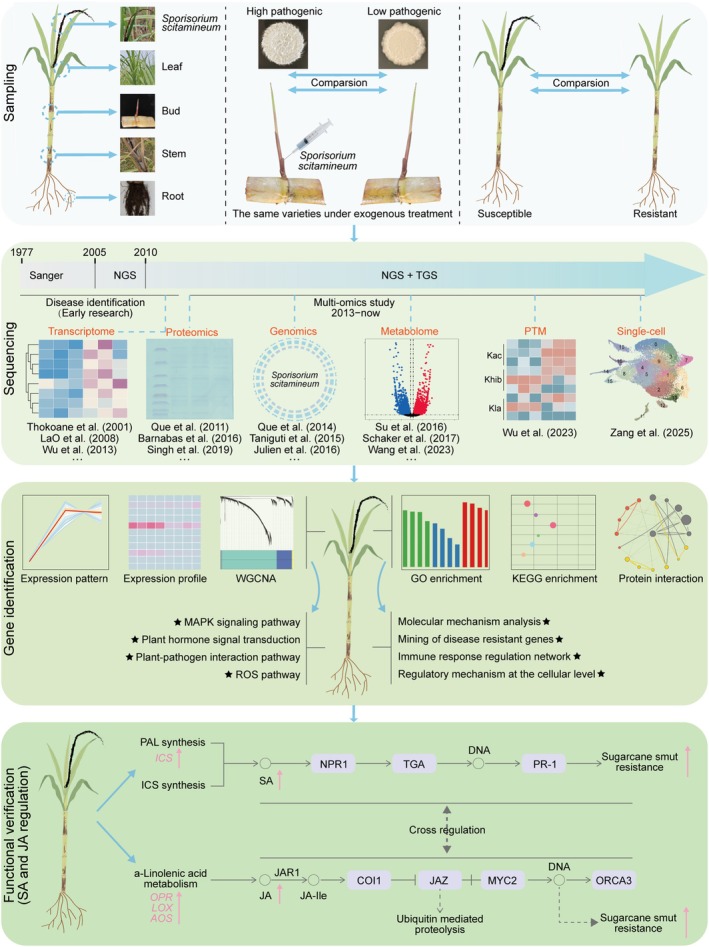
Schematic diagram of integrated multi‐omics analysis for sugarcane–*Sporisorium scitamineum* interactions. This schematic summarises how integrated multi‐omics technologies provide a systems‐level view of the sugarcane–*S. scitamineum* pathosystem. The approach simultaneously investigates both partners. For the pathogen, genomics identifies virulence determinants like effector genes. Time‐course transcriptomics and proteomics then reveal how these genes are dynamically expressed and translated during infection, elucidating strategies for host immune suppression and nutrient acquisition. For the host, genomics pinpoints potential resistance genes. Transcriptomics and proteomics profile the activation of defence pathways over time, such as the upregulation of phenylpropanoid genes (PAL) and salicylic acid (SA) biosynthesis (ICS). Metabolomics identifies and quantifies key antimicrobial compounds (e.g., phenolics, flavonoids). Post‐translational modification (PTM) proteomics (e.g., Kac, Khib, Kla) uncovers rapid regulatory mechanisms beyond transcription. Crucially, single‐cell transcriptomics resolves the cellular heterogeneity of the defence response, identifying which cell types (e.g., epidermis, vasculature) contribute specific defence functions. Bioinformatic integration (e.g., GO, KEGG enrichment analysis) synthesises these data layers into coherent biological narratives and regulatory networks. NGS/TGS: Next/third‐generation sequencing.

Multi‐omics technologies provide a paradigm shift beyond traditional, single‐layer approaches for systematically deciphering the mechanisms of sugarcane resistance to *S. scitamineum*. While conventional methods could identify individual resistance components, multi‐omics integration reveals the interconnected, multilayered nature of the defence system. By correlating multidimensional data from genomics to metabolomics, these approaches uncover previously inaccessible insights into how genetic variation orchestrates dynamic molecular and cellular responses to infection (Figure 3). This systems‐level perspective is crucial for moving from a descriptive list of resistance factors to a predictive understanding of the entire network, which directly informs rational breeding strategies.

#### 4.5.1 Genomics: Laying the Foundation for Host‐Pathogen Interactions

Genomic research serves as the core foundation for understanding host resistance and pathogen virulence. The *S. scitamineum* genome exhibits unique virulence‐associated characteristics, particularly in its clusters of genes encoding candidate secreted effector proteins (CSEPs), which are crucial for manipulating host immunity (Que, Xu, et al. 2014; Taniguti et al. 2015). From the host perspective, the resistance of sugarcane is closely related to genomic variations. Sugarcane genomes contain abundant resistance gene families, among which the NBS‐LRR class of resistance genes is a key member mediating disease resistance response. These resistance genes exhibit a clustered distribution pattern in the sugarcane genome and are often accompanied by copy number variations and sequence polymorphisms (Wu et al. 2022). Specifically, certain resistance genes can recognise effectors produced by the CSEP gene clusters of *S. scitamineum*, thereby triggering effector‐triggered immunity (ETI) (Ling et al. 2025; Ling et al. 2022). This process embodies a typical gene‐for‐gene interaction model. Such adaptive variations in the sugarcane genome do not occur randomly; the diversity of its resistance genes forms a dynamic response to the evolution of pathogen CSEP gene clusters. On one hand, sugarcane continuously expands the range of recognition for pathogen effector proteins through recombination, mutation and selective expression of resistance genes in its genome (Su et al. 2019). On the other hand, pathogens maintain their virulence through the rapid evolution of CSEP gene clusters (Dutheil et al. 2016). This coevolutionary relationship, elucidated by comparative genomics, provides a molecular explanation for the breakdown of race‐specific resistance in the field. Understanding this ‘arms race’ has direct practical implications: it argues for the deployment of polygenic or broad‐spectrum resistance in breeding programmes, rather than relying on single resistance genes which may be rapidly overcome by evolving pathogen populations. Furthermore, identifying the specific CSEPs recognised by sugarcane resistance genes enables the development of functional markers for precise selection of resistant genotypes.

#### 4.5.2 Transcriptome‐Proteome Synergy Reveals the Defence Cascade in Smut‐Resistant Sugarcane

Dynamic transcriptomic‐proteomic associations reveal that effective resistance relies on coordinated gene and protein regulation (LaO et al. 2008; Moleleki and Rutherford 2001; Wu et al. 2013). A clear contrast exists between resistant and susceptible varieties. Resistant varieties, such as YS1001, deploy a strong, multilayered defence. This includes the significant upregulation of key pathways like lignan/lignin biosynthesis and SA signalling, and a broader activation of defence genes at all stages, which provides robust immunity (Hu et al. 2024). Conversely, susceptible varieties like Co 97009 mount a weaker and ultimately unsuccessful response. Although they initially upregulate some hormone signalling, PR genes, and transcription factors, this response is not sustained. When facing highly virulent isolates, their PR gene expression is later suppressed by 60 days post‐inoculation (dpi). A concurrent upregulation of carbohydrate metabolism genes may instead support symptom development and pathogen success (Agisha, Ashwin, et al. 2022). For *S. scitamineum*, transcriptomics suggests that its *b*‐locus regulates carbon metabolism and stress responses, potentially via the Hog1 MAPK pathway, highlighting host–pathogen crosstalk (Agisha, Ashwin, et al. 2022). A significant uncertainty in such studies is distinguishing transcriptomic changes that are crucial for pathogenicity from those that are merely consequential to the host environment. The functional importance of many differentially expressed fungal genes, including those in the starch/sucrose pathway, for successful infection remains to be rigorously tested.

Proteomic analyses confirm defence‐related protein shifts in resistant sugarcane. For example, in resistant *S. spontaneum* , multi‐omics data link the upregulation of lignan/lignin biosynthesis genes to an increase in wall‐reinforcing metabolites like lignans. Similarly, iTRAQ‐based proteomics in resistant YC05‐179 detected a higher abundance of several defence proteins. These included PR proteins (PR1, PR2, PR5), peroxidases, and phenylpropanoid enzymes (4CL, CAD), which work together to enhance antimicrobial activity and lignin synthesis (Singh et al. 2019; Su, Xu, et al. 2016). Resistant lines also upregulate stress‐responsive proteins to mitigate oxidative damage, while susceptible lines downregulate defence‐related proteins. Integrated multi‐omics analysis reveals that resistance depends not just on having specific genes but on the precise coordination of a multilayered defence cascade. Transcriptomics identifies candidate genes, while proteomics and metabolomics confirm their functional output. This output includes the actual lignification of cell walls and the accumulation of antimicrobial compounds. This holistic view allows breeders to move beyond simple genetic markers. They can now select for varieties that possess the entire efficient regulatory network. For example, breeders could select parental lines that show strong, coordinated activation of the SA, phenylpropanoid and cell wall reinforcement pathways. This coordination is evidenced by their multi‐omics profiles. Selecting such lines could lead to progeny with more durable and robust resistance.

#### 4.5.3 Metabolomics and Rhizosphere Microbiome in Sugarcane Resistance to Smut

Metabolic differences are strongly associated with the distinction between smut‐resistant and susceptible sugarcane varieties. These differences are directly involved in the defence response by regulating the activation of key metabolic pathways and the synthesis of defence‐related substances (Chen et al. 2023; Schaker et al. 2017; Su, Xu, et al. 2016). A limitation of untargeted metabolomics, however, is its potential to miss critical metabolites present at very low concentrations but with high biological activity (e.g., certain phytohormones or signalling molecules), due to the limited dynamic range of detection platforms. Notably, shikimate pathway activation is a core metabolic event in resistant varieties: intermediates like shikimic and chorismic acid are more abundant, providing precursors for downstream compounds. As a primary‐secondary metabolism hub, it supplies carbon skeletons for phenylpropanoids (e.g., lignin, flavonoids) and biosynthesises amino acids (e.g., tryptophan, tyrosine), enhancing basal resistance via multi‐flux regulation (Hu et al. 2024; Wang et al. 2023). After smut infection, resistant varieties can quickly initiate secondary metabolic defence responses. Among them, the levels of lignin monomers increase significantly shortly after infection, forming a physical barrier by accelerating the deposition of lignin in cell walls to prevent the invasion and expansion of hyphae (Schaker et al. 2017). At the same time, the synthesis of flavonoids is also rapidly induced. These active substances can directly act on smut hyphae, exerting chemical defence functions by inhibiting their cell division and growth. This dual metabolic defence system of ‘physical barrier strengthening‐chemical toxin inhibition’ is an important metabolic basis for resistant varieties to resist pathogen infection (Nandakumar et al. 2021).

#### 4.5.4 Post‐Translational Modifications and Single‐Cell Analysis

Post‐translational modifications (PTMs) are key mechanisms for sugarcane to rapidly regulate protein function and cope with stress. Wu, Li, et al. (2023) conducted the first systematic identification of lysine acetylation (Kac), 2‐hydroxyisobutyrylation (Khib) and lactylation (Kla) in the sugarcane cultivar ROC22. They found that these modifications are highly enriched in core biological processes related to stress response, rather than being randomly distributed. Single‐cell transcriptome technology provides a new perspective for analysing the spatial heterogeneity of sugarcane stress responses and highlights the core role of cell type specificity in immune regulation. Zang et al. (2025) classified sugarcane bud cells into 10 major populations, including epidermal cells, vascular cells, and meristematic cells. They found that epidermal cells highly express genes such as *KCS* and *LTP* to activate the PTI pathway, forming the front line of defence; vascular cells, on the other hand, enhance JA signalling through genes related to lignin synthesis to transmit systemic defence signals. It also reveals the essential difference between the ‘active defence’ of resistant cultivars and the ‘passive response’ of susceptible ones. Despite its potential, single‐cell transcriptome in plants faces several challenges. First, dissociating plant cells requires enzymatic digestion. This process can itself induce a stress response, which may alter the transcriptome and confound genuine immune signals. Second, current protocols are often biased. They tend to capture more of certain cell types (e.g., parenchyma) while under‐representing others with tougher walls (e.g., sclerenchyma). This bias can lead to an incomplete picture of the cellular response. Finally, accurately annotating cell clusters is a major hurdle in non‐model plants like sugarcane. This is because comprehensive, cell type‐specific marker genes are often not available.

## 5 Current Management Strategies

### 5.1 Resistance Breeding

**FIGURE 4:**
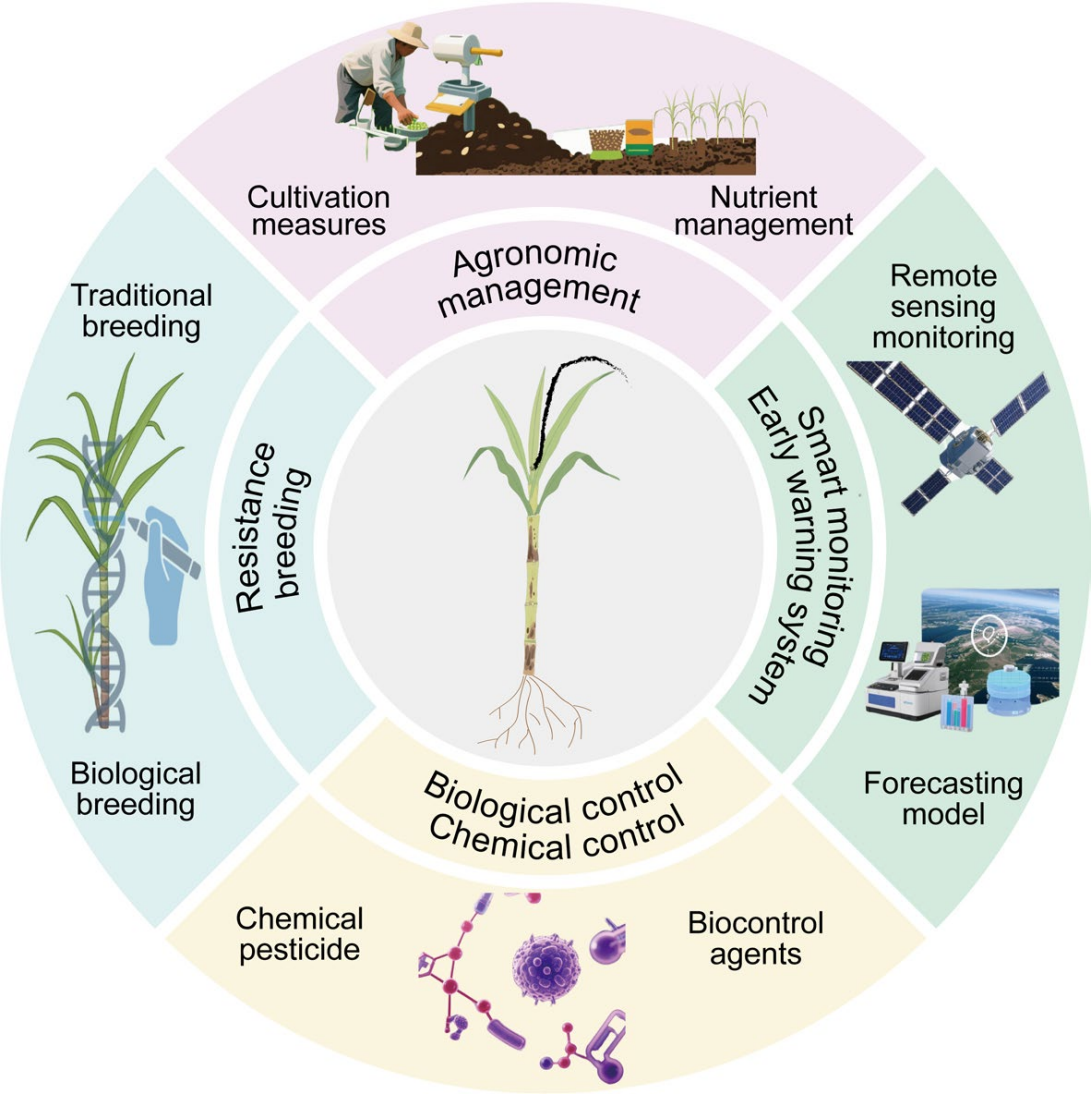
Comprehensive prevention and control strategies for sugarcane smut. Effective control of sugarcane smut relies on an integrated strategy combining multiple tactics. The foundation is Genetic Resistance, which involves breeding durable resistance into elite cultivars using modern tools like marker‐assisted selection and gene editing, informed by genomic and multi‐omics research. Cultural Practices are crucial for reducing initial pathogen inoculum and creating an environment less favourable to disease, including the use of healthy planting material (pathogen‐free setts), crop rotation, and field sanitation. Biological Control uses beneficial microorganisms to antagonise the pathogen or prime plant defences. Chemical Control involves the targeted application of fungicides for sett treatment or field application, though with careful consideration to avoid environmental and resistance issues. Finally, Smart Agricultural Systems leverage technologies like remote sensing for early detection of disease foci and predictive modelling based on meteorological data to provide timely warnings and enable precision management. The synergy of these components forms a robust defence system that minimises economic losses.

Current resistance breeding against sugarcane smut integrates conventional methods with modern biotechnology (Figure 4). Conventional approaches involve screening hybrid progeny for resistant individuals, followed by pedigree selection and multigenerational breeding to develop stable cultivars. Medium‐to‐highly resistant varieties like GT42 and YZ08‐1609 have significantly reduced disease losses in production. Recurrent selection enhances the frequency of resistance genes within populations through multiple rounds of intercrossing resistant individuals (Wu, Li, et al. 2024; Wu, Li, et al. 2025). Modern biotechnological breeding leverages transgenic technology and genome editing (Iqbal et al. 2021). Recent breakthroughs include the development of highly smut‐resistant transgenic sugarcane lines by using host‐induced gene silencing (HIGS) technology to target pathogen virulence genes (Wu, Qiu, et al. 2025). Furthermore, convergent crosses using wild germplasm resources and highly resistant cultivars can produce new germplasm combining high sucrose content with robust resistance (Wu, Li, et al. 2025).

### 5.2 Agronomic Management

Agronomic management focuses on source control and ecological regulation (Figure 4). Seedling treatment is crucial for blocking primary infection: soaking seed cane in 52°C hot water for 30 min effectively inactivates teliospores (Bhuiyan et al. 2012). The use of pathogen‐free tissue‐cultured seedlings further minimises pathogen transmission risks. Optimised cropping systems include rotation with non‐host crops (e.g., rice, maize, sweet potato) to disrupt the survival cycle of the pathogen, and shortening ratoon cycles and promptly removing infected plant debris (via deep burial or burning) to reduce soil spore accumulation. Additionally, nutrient management requires balanced nitrogen (N), phosphorus (P) and potassium application: excessive N fertiliser promotes succulent growth and lowers resistance, whereas adequate P enhances disease resistance; and sufficient basal fertilisation combined with improved drainage and irrigation promotes robust cane growth. In rainfed sugarcane areas, soil moisture conservation is essential to prevent drought conditions that exacerbate spore accumulation.

### 5.3 Chemical and Biological Control

Chemical control primarily relies on systemic fungicides like tebuconazole and benzoyl acrylate + azoxystrobin (Figure 4). Application during the tillering stage effectively suppresses mycelial growth and spore germination, but strict management of application frequency and dosage is critical to prevent fungicide resistance development. Biological control exploits the multi‐faceted antagonistic mechanisms of beneficial microbes: *Trichoderma harzianum* and *Bacillus subtilis* suppress the pathogen through nutrient competition, parasitism and ISR (Aslam et al. 2023). Field trials have confirmed their stable efficacy and high environmental compatibility (Viswanathan and Malathi 2019). Green production bases are recommended to prioritise biological agents, supplemented by rotational use of chemical pesticides.

### 5.4 Smart Monitoring and Early Warning Systems

Smart systems integrate multisource data and technological synergy for precise disease control (Figure 4). Remote sensing (satellite/UAV imagery) combined with AI‐based image recognition enables rapid mapping of disease foci and severity assessment (Kunduracıoğlu and Paçal 2024; Moriya et al. 2023). Molecular diagnostics (e.g., PCR) detect pathogens in plants or soil even before symptom expression, enhancing early warning capabilities. Furthermore, predictive models integrate meteorological data, soil conditions, sugarcane growth status and historical disease incidence records. These models effectively forecast the timing, epidemic trajectory and severity of disease outbreaks, enabling growers to implement preventative measures proactively (Wang, Wu, et al. 2025; Wang, Wu, et al. 2024). This significantly improves the scientific rigour and effectiveness of disease management.

## 6 Emerging Challenges and Future Directions

Current research on sugarcane smut disease faces three intertwined challenges. The primary challenge is the rapid adaptive evolution of the pathogen. Continuous mutation generates new virulent races capable of overcoming existing resistance gene defences, leading to rapid resistance breakdown in host cultivars and increasing difficulty in the discovery of new resistance sources. Secondly, climate change poses a significant threat. Rising atmospheric CO_2_ concentrations may alter sugarcane physiological metabolism and the synthesis of defence compounds. Increasingly frequent extreme rainfall or drought events disrupt pathogen dispersal environments and weaken sugarcane vigour, making disease occurrence more unpredictable. Thirdly, sustainable control faces multiple pressures: environmental pollution and non‐target hazards from long‐term reliance on chemical fungicides, restrictions on chemical inputs driven by expanding organic markets, and the problem of rapid ‘resistance erosion’ associated with single‐gene resistance deployment. These factors collectively constrain the economic and ecological sustainability of control strategies. Future research must focus on highly advanced, cross‐disciplinary technological breakthroughs:

1. AI‐Driven Precision Disease Management: Moving towards deep AI integration, machine learning will fuse high‐throughput climate monitoring, real‐time pathogen genomic variant tracking and large‐scale crop phenotypic data to build dynamic forecasting models. Integrated with intelligent sensing networks (drones, Internet of Things [IoT]), this will enable precise identification of infection hotspots and intelligent control decision‐making.
2. Intelligent Multi‐Omics Guided Resistance Breeding: This integrates genomics (mining novel resistance from wild relatives), transcriptomics (deciphering defence signalling networks), metabolomics (screening key resistance metabolite markers) and epigenomics. AI will accelerate the design and breeding of breakthrough varieties with broad‐spectrum and durable resistance, fundamentally solving the problem of resistance being readily overcome
3. Precision Bi‐Directional Gene Editing: Advancing towards more precise targeted control. On one front, CRISPR‐Cas9 and similar tools will edit key sugarcane susceptibility genes or enhance endogenous immune pathways. On another front, research will explore designing specific gene drive systems or RNA interference (RNAi)‐based delivery strategies to achieve targeted silencing or population‐level suppression of key virulence genes within the pathogen or its vectors.
4. Synthetic Biology Empowered Green Control: Innovations will rationally design engineered synthetic microbial consortia, reprogramming their metabolism to enhance rhizosphere colonisation efficiency and the sustained production of novel anti‐smut compounds (e.g., novel antimicrobial peptides, lytic enzymes). Development will focus on environmentally responsive, biodegradable nanocarriers for the spatiotemporally precise controlled release of fungicides/biopesticides. Novel plant vaccine concepts (protein/nucleic acid elicitors) will be explored to efficiently induce systemic resistance. Concurrently, metabolomics‐guided manipulation of root exudates will optimise the structure of rhizosphere and endophytic antagonistic microbial communities.

## 7 Conclusions

Taken together, multi‐omics advances have profoundly elucidated the molecular arms race between *S. scitamineum* and sugarcane, revealing sophisticated pathogenic strategies and layered host defence mechanisms. Moving forward, we recommend a concentrated research strategy that translates these foundational insights into practical applications. Key priorities should include developing AI‐driven models for precise disease forecasting, applying integrated multi‐omics to identify robust resistance traits for breeding programmes, and pioneering ecofriendly interventions such as precision gene editing and engineered microbial consortia. This concerted focus on predictive, durable and sustainable solutions is critical for safeguarding global sugarcane production.

## Author Contributions

**Zhen Zeng:** funding acquisition, writing – original draft, writing – review and editing, visualization. **Qibin Wu:** funding acquisition, writing – original draft, writing – review and editing, project administration, supervision. **Dongjiao Wang:** conceptualization, resources. **Wanying Zhao:** conceptualization, resources. **Yuanyuan Zhang:** conceptualization, resources. **Tingting Sun:** conceptualization, resources. **Wankuan Shen:** conceptualization. **Youxiong Que:** funding acquisition, conceptualization, writing – original draft, writing – review and editing, project administration, supervision.

## Funding

This work was supported by Chinese Academy of Tropical Agricultural Sciences for Science and Technology Innovation Team of National Tropical Agricultural Science Center (CATASCXTD202402), Science and Technology Major Project of Guangxi (Guike AA23073001), Project of State Key Laboratory for Tropical Crop Breeding (SKLTCBBSH202504, NKLTCB‐HZ04, NKLTCBCXTD24, NKLTCBCXTD38, and SKLTCLHZD202502), National Natural Science Foundation of China (32301873 and 32460497), Hainan Provincial Natural Science Foundation High‐level Talent Program (325RC817), Central Public‐interest Scientific Institution Basal Research Fund (163002025013 and 1630052025021), and China Agriculture Research System of MOF and MARA (CARS‐17).

## Conflicts of Interest

The authors declare no conflicts of interest.

## Data Availability

The data that support the findings of this study are available from the corresponding author upon reasonable request.
